# Items

**Published:** 1897-03

**Authors:** 


### Items.

A COURSE IN BACTERIOLOGY.—Dr. Frank J. Thornbury desires to announce a course in bacteriology, beginning on the 18th of February, to be held in the bacteriological laboratory of the University of Buffalo. It will be limited to ten men, and composed chiefly of physicians. The fee is $10, and the hours for work 8 to 10 P. M., three evenings each week for six weeks. The course will comprise the preparation of culture media, general bacteriological technique, a study of each of the standard nonpathogenic organisms and then the pathogenic ones, animal experiments being used in illustrating the etiological characters of the latter. The molds will be studied, and the examination of air, earth, water and milk will be taken up in detail.

Special attention will be given to subjects of practical clinical importance, as the examinations of the nasal and pharyngeal secretions for the influenza bacillus, the serum diagnosis of typhoid fever, the finding of the faden bacillus in cases of carcinoma, and the culture and staining of the typhoid, diphtheria, influenza, tubercle, and glanders bacillus and the gonococcus and cholera spirillum; also the isolation of typhoid bacillus from water. The technique for testing the relative merits of disinfectants will be elaborated upon. Some immunising experiments in typhoid fever, cholera and diphtheria will be made.

The system originally outlined by Professor Koch for courses in bacteriology in the Hygienic Institute, Berlin, will be followed.

---

J. PIERPONT MORGAN, according to a recent notice in the newspaper press, has given the munificent sum of $1,000,000 to the New York Lying-in Hospital. Mr. Morgan's letter said, in substance, that he believed a suitable building should be erected on the property now owned by it and that he had had plans prepared to erect the same at his own expense, the building to cost about $1,000,000. The only condition attached to the gift is that Mr. Morgan shall have assurance that the hospital will have funds enough to continue its work after the building is completed. The gift was promptly accepted by the board, which is confident that it can satisfy the donor as to the one condition. The board expect the new building will be begun shortly and, when completed, it will be one of the finest hospitals of its kind in the world.

---

Too many men of the medical profession, it is feared, are apt to pin their faith to a drug that bears a long chemical name, manufactured perhaps in Germany, which is considered a guarantee of its purity and ethical property. The fact is, most of such drugs are patented, hence are irregular from an ethical standpoint. It is a satisfaction, however, to know that many American manufacturing chemists are producing pure medicines at a less cost, and with equally or, perhaps, more certain results in their therapeutic application. Attention is invited, in this connection, to the advertisement of the Charles Roome Parmele Company in this issue of the Journal.

---

As successor to the famous physiologist, Du Bois-Reymond, head of the Physiological Institute in Berlin and professor of physiology in the University, Professor Hermann Munk will probably be chosen, since Herr von Kries-Freiburg, to whom the place was offered, has declined it. Professor Munk, who is fifty-eight years old, was a pupil of Du Bois-Reymond, and is a member of the Berlin University faculty and professor and director of the physiological laboratory of the Veterinary High School in Berlin. He has been a member of the Academy of Science since 1880. His selection meets with warm approval among the scientists of Berlin.

---

Dr. Herbert U. Williams will commence his spring class in examination of blood, sputum, and the like, in a few weeks. The course is limited to a small number of physicians and will take place evenings at the University of Buffalo.

---

The Diet in Chronic Diarrhea of Infants.—Angel Money (*Treatment of Diseases of Children*) says if the child be under one year of age the diet must not go beyond the range allowed at that period of life. Barley water and whey, perhaps milk according to circumstances and Mellin's Food, raw meat juice, white of egg or yellow of egg with water, mutton broth, weak beef tea, is a sufficient list of foods. It is generally asserted that animal broths are deleterious in diarrhea, used in small quantities cold. He has not noticed any prejudicial effect, and certainly the number of stools has not appeared to be increased.

